# Factors influencing adolescent experimental and current smoking behaviors based on social cognitive theory: A cross-sectional study in Xiamen

**DOI:** 10.3389/fpubh.2023.1093264

**Published:** 2023-03-22

**Authors:** Manzhi Lin, Meijie Chu, Xian Li, Honghao Ma, Zhiwei Fang, Li Mao, Pengjun Wang, Tianmu Chen, Yi-Chen Chiang

**Affiliations:** ^1^Xiamen Huli District Center for Disease Control and Prevention, Xiamen, China; ^2^State Key Laboratory of Molecular Vaccinology and Molecular Diagnostics, School of Public Health, Xiamen University, Xiamen, China; ^3^School of Management, Xuzhou Medical University, Xuzhou, China

**Keywords:** experimental smoking, current smoking, adolescents, social norm, positive outcome expectation, anti-smoking self-efficacy, anti-smoking policy attitudes

## Abstract

**Introduction:**

China has the largest youth population in the world. To better implement the Smoke-free School Initiative, this study aims to examine the protective and risk factors for different smoking behaviors (never smoked, experimental smoking, and current smoking) among school adolescents based on social cognitive theory.

**Methods:**

This research was a secondary analysis of a cross-sectional survey of middle schools in Huli District of Xiamen, China. The final sample consisted of 1937 participants with an average age of 15.41 (SD = 1.64). Descriptive statistics were used to summarize the sociodemographic characteristics of the sample. Multivariate multinomial logistic regression analysis was performed using four models.

**Results:**

Of the respondents, 1685 (86.99%) were never smokers, 210 (10.84%) were experimental smokers, and 42 (2.17%) were current smokers. Social norms, positive outcome expectations, anti-smoking self-efficacy, and attitudes toward control tobacco policies were associated with adolescents' smoking behaviors. The number of smoking family members, classmates smoking, the perception that smoking is cool and attractive, and attitudes toward control tobacco policies were the predictors of current smoking behavior (*p* < 0.05). In contrast, friends smoking and individual and social relationship motivation were associated with only experimental smoking (*p* < 0.05).

**Discussion:**

The relationship of social norms, positive outcome expectations, anti-smoking self-efficacy, and attitudes toward control tobacco policies varied across smoking behaviors. Family, school, society and the government need to cooperate in prevention and intervention programs for adolescent smoking. The relationships between these factors and adolescents' different smoking behaviors needs to be further verified.

## 1. Introduction

Smoking has become a public health problem of great concern worldwide. Smoking causes serious harm to the respiratory system and cardiovascular system and accelerates the occurrence of chronic diseases in adulthood. The China Global Tobacco Youth Survey (2019) shows that China is the world's largest producer and consumer of tobacco (1). Cigarette smoking is rising among Chinese adolescents and poses a significant public health concern (2, 3). Behind the 6.9% of adolescents using tobacco, 19.9% of adolescents have tried tobacco products, and 82.3% of attempts to smoke occurred before the age of 13, most attempts occurred in primary school (4). The result also shows that the exposure rate of secondhand smoke among middle and high school students in China is 63.2 and 72.0%, which is still relatively serious. And the proportion of junior high school and general high school students who were not rejected because of age in the last time they bought cigarettes was as high as 76.5, 87.6% respectively (5). Studies have shown that from a regional point of view, the frequency of smoking among young people in suburban areas is higher than that in municipalities directly under the central government and provincial capitals. Compared with municipalities directly under the central government and provincial capitals, there are significant differences in social culture, economic development level and civilization level in suburban areas, indicating that regional factors have a significant impact on youth tobacco use, and adolescents in areas with lower socioeconomic levels are more likely to use addictive substances (6). The Chinese data also showed that economic expansion reduces men's smoking amount (7). Smoking behavior may be adopted to counter physical or psychological pressure not directly related to change in current income level. Xiamen, located on the southeast coast of China, is one of the first special economic zones (8), the urbanization rate of permanent residents reached 90.1% (9). Xiamen's economic growth is in line with the growth rate of China. This study discussed some factors associated with adolescents' smoking behavior in Xiamen, China, which would provide some information for the national control of tobacco.

Smoking among adolescents in China is dominated by males, but the smoking rate of female adolescents has shown an upward trend. Reducing smoking among adolescents is of great significance for overall tobacco control work (10). Experimentation with smoking is a critical step to becoming a regular smoker (11), and “refusing the first cigarette” is an important measure for youth to control smoking (12). Some studies have shown that trying to smoke increases the risk of smoking by 3–6 times in middle school students (13, 14), and adolescents who try to smoke are more likely to become new smokers (15). Smoking experimentation and initiation rates increase in adolescence. The age of “trying to smoke” among adolescents is gradually occurring earlier, and the prevalence shows a clear upward trend with age. The appropriate strategies for reducing smoking behaviors, including experimental smoking and current smoking, need to be further discussed.

### 1.1. Theoretical background

Social cognitive theories (SCT), which focuses on cognitive factors (such as belief, memory, expectation, motivation and self-reinforcement), are useful for explaining health behaviors such as physical activity and unhealthy behaviors such as smoking (16). SCT provides a comprehensive and well-supported conceptual framework for understanding the factors that influence human behavior. But its greater significance has come from its application to the design of interventions to meet important practical challenges in public health. Previous evidence showed that one telephone counseling service was offered by the American Cancer Society (ACS) and they help smokers quit by providing guidance in self-regulation. The theories propose that the human learning process is an observational learning process that can not only rely on individual action, but also enable learning by observing and imitating models encountered by others in the environment and acquiring information (17). A system called triadic reciprocal determinism is a focus of social cognitive theories; this system involves the interaction between individual factors (self-efficacy, outcome expectation, knowledge, etc.), physical and social environmental factors (peer influence and social norms, resources, behavioral outcomes, policies, and physical settings), and behavior (individual actions, choices, and verbal statements) (18). Bandura emphasized that cognitive determinants, including outcome expectations and self-efficacy, operate as determinants of behavior (18). Adolescents are in a critical period of extensive individual cognitive development (19); they tend to be curious and show a wide range of interests and emulate their esteemed peers and non-parent adults (20). Thus, numerous previous studies of adolescents' behaviors have mentioned SCT (21–23). There is evidence that theory-based interventions are more effective than theory-free approaches (24–26).

SCT states that the initiation and persistence of behaviors are determined primarily by outcome expectations and self-efficacy (27). Smoking-related cognitions (including self-efficacy, social norms and attitude) predict smoking intentions and smoking behavior among adolescents (28, 29). Among smoking-related cognitions, self-efficacy is the best predictor of adolescents' smoking behaviors. A positive attitude toward smoking or adolescents' perceptions of the social influence of smoking predict an increased risk of adolescents smoking (30). The family and the school are the closest social contexts to developing adolescents, making their relationships with adolescents' behaviors a key object of analysis. School-based programs to prevent tobacco use can make a substantial contribution to reducing the percentages of adolescents who smoke. School-based programs included the introduction of policies, creating a friendly environment, health education, home school collaboration, and advocating for the whole society to take action in China (31). The “Smoke-free School” campaign focuses on schools formulating clear anti-smoking policies and correcting students' attitudes toward tobacco control policies. Thus, this study discusses the influencing factors of experimental and current smoking among adolescents through SCT.

### 1.2. The social and individual level influencing factors of smoking among adolescents

#### 1.2.1. Social norms

It has been widely established that the behavior of adolescents is often influenced by peers, parents and normative beliefs (32–36). Social norms are the rules, values, or standards shared by the members of a social group that define the appropriate, expected, or desirable attitudes and behaviors in matters pertaining to that group (37). In other words, social norms are implicit codes of conduct that provide a guide to appropriate behavior (38). Social norms include descriptive and injunctive norms (39). Descriptive norms are adolescents' perceptions about the frequency of certain risky behaviors around them, while injunctive norms are the real beliefs about the approval of behaviors (40). Using social norms to understand the environment and interpersonal influences to change behavior can be more effective than focusing on individuals to change behavior (41). Extensive research shows that social norms are critical determinants of adolescent risky health behaviors (42, 43). Social norms toward smoking are a key concept in tobacco control policy and research. Social norms of parents' and close friends' smoking behavior appeared to be consistent predictors of youth smoking initiation (44). Previous studies have indicated that perceived disapproval of smoking may reduce smoking behaviors (45, 46). Individuals with permissive smoking norms exhibit more smoking (OR 1.34, 95% CI 1.03–1.74), particularly among those with no history of smoking (47), showing that adolescents who perceive cigarette use as more prevalent and acceptable are more likely to initiate tobacco use (48). Social norm interventions provide correct information about peer group norms to correct misconceptions about norms (49, 50). Thus, the social norm of smoking is an important influencing factor for adolescent smoking and for experimentation with smoking.

#### 1.2.2. Positive outcome expectations

Adolescent smoking behavior continues to be a challenging issue in large part due to outcome expectations. Among cognitive determinants, outcome expectations, which involves the anticipated consequences (positive or negative) of behaviors (18, 51, 52), is emphasized as a determinant of behavior. The analysis of behavior in terms of expected outcomes has a long history in psychology, and this approach has been applied to several diverse fields (53). These expectations have been studied extensively in adolescent behavioral medicine and have been found to influence a variety of health behaviors, including smoking, alcohol consumption and weight management (54–60). Adolescents who smoke may have multiple positive outcome expectations about their perceived benefits, such as social confidence (58), stress reduction (61) and weight control (62). According to SCT, individuals who expect more positive outcomes from smoking are more likely to smoke because they mistakenly believe that smoking provides more benefits (63). In a longitudinal study, Wahl et al. found that smoking-related outcome expectations, such as negative affect management (i.e., makes me feel good) and boredom relief, may influence smoking initiation among eighth and tenth graders (57). Depending on the severity of the health behavior, along with the individual's personality traits, outcome expectations may be a large potential challenge to behavioral change.

#### 1.2.3. Anti-smoking self-efficacy

Anti*-*smoking self-efficacy is the ability of adolescents to remain non-smoking and refuse to smoke (64). Previous studies have shown that adolescents with lower anti-smoking self-efficacy exhibit more smoking behavior (65). A high level of anti-smoking self-efficacy not only may reduce individuals' attempts to smoke or lead them to stop smoking (66, 67), but also may act as protective factor against future smoking behavior (68, 69). In addition, a high level of anti-smoking self-efficacy plays a very important role in individuals resisting external factors such as parental and peer influence (70, 71), advertising and pro-smoking media (72, 73) and social atmosphere (74). The age at which adolescents first smoke may predict the number of cigarettes smoked and dependence in the near future (75). Thus, the self-efficacy of refusing the first cigarette is an important measure for youth to control smoking. The important aspect of refusing smoking for self-efficacy among adolescents needs to be explored.

#### 1.2.4. Attitudes toward control tobacco policies

Smoking ban policy is one of the most important controllable aspects of social environment (76). China became a party to the WHO Framework Convention on Tobacco Control (WHO FCTC) in 2006. China does not have one comprehensive tobacco control law, but several national laws and regulations that legislate tobacco. These national laws included (1) Prohibit smoking in at least 28 indoor public places, including medical facilities, restaurants, bars, and most public transportation. (2) Prohibit all film, television, radio, in newspapers and magazines advertising. Point of sale, online advertising, and sponsorship is permitted. (3) Require text-only warnings that, at maximum, cover 30% of the pack. Tobacco companies can create their own warning labels as long as they meet minimum criteria. (4) Increased the tax on tobacco as a percent of retail price to over 60%. (5) Allow sub-national regulations that are stricter than the national law, including those that restrict tobacco advertising, promotion, and sponsorship (TAPS) outdoors. From 2004 to 2014, the smoke-free policy was applied across more than a dozen cities in China. The International Tobacco Control (ITC) on China Survey found high levels of support in China for stronger smoke-free policies, even among smokers. This situation provided support for adolescents' smoking cessation. Some studies found that public health policies appear to have decreased the overall prevalence of adolescents' smoking but with only a weak effect (77). Individual policy literacy may be a critical factor in improving preventive care and reducing health disparities (78). Some scholars have analyzed the relationship between attitudes toward the policy and social cognitive determinants of smoking (79). Regarding adolescent smoking behavior, if smokers have low policy literacy, they are at greater risk of smoking continuation. This may be due to their misconceptions and wrong attitudes toward tobacco control policies.

However, a great deal of research has explored the influencing factors of smoking behaviors among adolescents. It is important to focus on two processes of smoking that adolescents may be engaged in: experimental smoking and current smoking. Different predictors may explain these two smoking processes, and thus the role of several factors may differ between them. Few studies have analyzed the relationships of multiple internal and external factors with adolescents' different smoking behaviors in Xiamen, China. This study aimed to investigate the protective and risk factors for different smoking behaviors (current smoking and attempt at smoking) among school adolescents through SCT in Xiamen city, China.

## 2. Methods

### 2.1. Participants

The Xiamen Center for Disease Control and Prevention (CDC) conducted a tobacco survey among primary and middle school students in Xiamen city (80, 81). Data in this study were derived from a 2017 cross-sectional survey of cigarette smoking among junior Chinese middle school students in Huli District, Xiamen, which is a part of this persistent project. Huli District is located in the north of Xiamen and belongs to the central city of Xiamen, which is the birthplace of Xiamen Special Zone and has strong regional economic vitality, as well as being the center of the national information technology industry base. According to the number of registered middle schools in Huli District, Xiamen city, there are 10 middle schools. The total sample size of middle school students in this study was 1938, and due to excluding one student who skipped too many items on the questionnaire, the total effective sample size was 1937, for a response rate of 99.9%. These 1937 middle school students aged 11–18 years old were from four randomly selected middle schools. Informed consent was obtained from all the participants and their guardians in this study. The method of investigation was face-to-face interviews.

### 2.2. Measurements

The measurement instruments involved in this study were guided by the Global Youth Tobacco Survey (GYTS) (82) and adapted to the reality of adolescent smoking in China (83, 84).

#### 2.2.1. Smoking behavior

According to the definition of smoking standards recommended by the WHO (85), respondents indicating that they had smoked at least a complete cigarette in the past 30 days were defined as current smokers. Smoking behavior was a dependent variable in this study, including the three statuses of never smoked, experimental smoking, and current smoking.

#### 2.2.2. Social norms

SCT posits that portions of an individual's knowledge acquisition can be directly related to observing others within social contexts. Social contexts influence adolescents smoking behavior is by providing role models and by setting social norms concerning smoking (86). The key persons influencing the social norms of adolescent smoking behavior in this study were family members, teachers, and peers. For family members, the students were asked whether their fathers, mothers, grandfathers, or grandmothers who lived with them smoked, the number of family members who smoked, and the parents' attitudes toward their children about smoking. The students were also asked whether their school administrators, male teachers, female teachers, homeroom teachers, and school welfare officers' smoke. The students were asked whether their classmates (same class, same grade, or same school) or friends smoke. The split-half reliability was 0.672.

#### 2.2.3. Positive outcome expectations

Positive outcome expectation is the anticipated positive consequences of a behavior (18). It is measured by six items by asking students whether they agree that (1) smoking is a personal choice and outsiders should not interfere because smoking does not affect others; (2) smoking is a sign of psychological maturity and a sign of career success; (3) the fact that many people in society smoke indicates that the benefits of smoking outweigh the disadvantages; (4) smoking is cool and attractive; (5) smoking is a necessity for social communication; and (6) smoking can refresh and relieve boredom. Responses are scored as “1 = Yes, 0 = No.” The split-half reliability was 0.612.

#### 2.2.4. Anti-smoking self-efficacy

Anti-smoking self-efficacy is the judgment of one's capability to exhibit anti-smoking behavior (87). It included three items in this study. (1) What do you do when someone smokes in front of you? The responses are “1 = smoke with them, 2 = it doesn't matter, 3 = avoid or ask them to smoke elsewhere, 4 = advise them not to smoke.” (2) When someone hands you a cigarette, what is your attitude? The responses are “1 = accept it calmly, 2 = want to refuse but embarrassed, 3 = refuse.” (3) What is your attitude when a stranger sitting next to you smokes in a place with a no-smoking sign? The responses are “1 = try to endure, 2 = keep away from the person, 3 = discourage or stop the person.” The split-half reliability was 0.660.

#### 2.2.5. Attitudes toward control tobacco policies

It included four items: (1) evaluation of the behavior of someone smoking in public; (2) moral evaluation of middle school students who smoke; (3) attitudes toward smoking being banned in schools; and (4) attitudes toward adolescents smoking being banned. The total score ranges from 0 to 4, with higher scores indicating a better attitude toward control tobacco policy.

### 2.3. Data analysis

Statistical analyses were performed using SAS version 9.4. Descriptive statistics about the distribution of variables are presented as frequency distributions and percentages, means and standard deviations. Multivariate multinomial logistic regression analysis was performed using four models to assess the influencing factors of adolescents' experimental smoking and current smoking behaviors. Model 1 clarifies the impact of social norms on adolescent smoking based on controlling for gender. Model 2, Model 3, and Model 4 successively add positive outcome expectations, anti-smoking self-efficacy, and attitudes toward control tobacco policies based on the previous model. Statistical assumptions for multivariate logistic analysis and the checkout of this study were provided in the Supplementary File.

## 3. Results

### 3.1. Sample characteristics

The final sample consisted of 1,937 participants with an average age of 15.41 (SD = 1.64) (Table 1). The majority of the adolescents were boys (1,055, 54.58%). Approximately 1,146 (59.13%) students were in 8th grade, and 792 (40.87%) students were in 10th grade. The proportions of students perceiving their fathers, mother, grandfather, grandmother smoking were 51.86, 1.82, 34.35, 2.45%, respectively. The adolescents reported the number of family members who smoke was 0.94 (SD = 0.95). In addition, injunctive norms of perceived disapproval of smoking among parents was 2.93 (SD = 0.30). In the school environment, students' perceived prevalence rates of smokers were high in male teachers (49.97%), and school leadership (35.79%). Approximately 5.01% of the participants perceived their female teachers smoking. Approximately 12.43% of participants who perceived their classmates smoking, and the proportion of participants who perceived their friends smoking was 8.45%. In terms of the positive outcome expectation, approximately 13.90% of the adolescents held the opinion that smoking is a social need, and 19.69% held the opinion that smoking can refresh and relieve boredom. In terms of anti-smoking self-efficacy, the scores of refusing skills or negative attitudes to the scenarios of “Someone smokes in front of you,” “Someone offers you a cigarette” and “The stranger smokes in a no smoking area” were 3.32 ± 0.71, 2.85 ± 0.43 and 2.50 ± 0.61, respectively. Moreover, the score of attitudes toward control tobacco policies was 2.39 (SD = 0.70).

**Table 1:** Characteristics of the study sample in Huli District, Xiamen, China (*n* = 1,937).

**Variable**	***n* (%)/Mean ±SD**
**Age**	15.41 ± 1.64
**Grades**	
8th	1,146 (59.13)
10th	792 (40.87)
**Gender**	
Boy	1,055 (54.58)
Girl	878 (45.42)
**Social norm**	
**Family members who smoke**	
Father	1,003 (51.86)
Mother	35 (1.82)
Grandfather	662 (34.35)
Grandmother	47 (2.45)
Number of family members who smoke [0-3]	0.94 ± 0.95
Parents' negative attitudes [1–3]	2.93 ± 0.30
**Teachers who smoke**	
School leadership	582 (35.79)
Male teachers	828 (49.97)
Female teachers	85 (5.01)
Head teachers	92 (5.21)
School support staff	362 (22.47)
**Peers who smoke**	
Classmates	238 (12.43)
Students in the same grade	208 (10.86)
Schoolmates	183 (9.58)
Friends	163 (8.45)
**Positive outcome expectation**	
A personal choice	102 (5.27)
A sign of maturity and success	91 (4.70)
Advantages greater than disadvantages	88 (4.55)
Cool and attractive	114 (5.89)
Social need	269 (13.90)
Refresh and relieve boredom	381 (19.69)
**Anti-smoking self-efficacy**	
Avoid smoking when someone smokes in front of you [1–4]	3.32 ± 0.71
Refusing a cigarette offered by others [1–3]	2.85 ± 0.43
Negative attitude to a stranger smoking in a no smoking area [1–3]	2.50 ± 0.61
**Attitudes toward control tobacco policies [2–9]**	2.39 ± 0.70
**Smoking behaviors**	
Non-smoking	1,685 (86.99)
Experimental smoking	210 (10.84)
Current smoking	42 (2.17)

Data are presented as the mean ± SD or frequency and percentage. The minimum score and maximum score of every sociopsychological variable are present in brackets.

### 3.2. Adolescents' self-reported smoking behaviors

The majority of the adolescents were non-smokers (86.99%), and ~2.17% of the adolescents smoked at present. Furthermore, ~10.84% of the young people were experimental smokers who have tried smoking in the past but do not smoke now.

### 3.3. Multivariate logistic regression analyses of influencing factors of adolescents' smoking behaviors (experimental smoking vs. non-smoking)

Table 2 indicates the results of multivariate logistic regression analyses of the influencing factors of adolescents' smoking behaviors (experimental smoking vs. non-smoking). In Model 1, we analyzed the associations between social norms and adolescents' smoking behaviors. Parents' negative attitudes were a protective factor against experimental smoking (OR = 0.39, 95% CI: 0.24–0.62, *P* < 0.001). The adolescents who reported female teachers and friends smoking were much more likely to be experimental smokers (OR = 2.72, 95% CI: 1.27–5.83, *P* = 0.010; OR = 3.24, 95% CI: 1.85–5.68, *P* < 0.001) than the adolescents who were not exposed to female teachers and friends smoking. Based on Model 1, positive outcome expectations were added to Model 2. The adolescents who reported that “Smoking is a social need” and “Smoking can refresh and relieve boredom” were more likely to be experimental smokers (OR = 1.68, 95% CI: 1.01–2.80, *P* = 0.045; OR = 2.86, 95% CI: 1.86–4.39, *P* < 0.001). We added anti-smoking self-efficacy variables in Model 3 based on Model 2. The adolescents who had more anti-smoking self-efficacy, such as skills to avoid smoking when someone smoked in front of them and to refuse a cigarette offered by others, were less likely to attempt to smoke (OR = 0.66, 95% CI: 0.50–0.86, *P* = 0.002; OR = 0.62, 95% CI: 0.41–0.93, *P* < 0.019). Attitudes toward control tobacco policies was not a significant predictor of experimental smoking behavior among adolescents (Model 4).

**Table 2:** The influence of smoking social norms, positive outcome expectations, anti-smoking self-efficacy and attitudes toward control tobacco policies on adolescents' smoking behaviors (experimental smoking vs. non-smoking).

**Variable**	**Model 1**	**Model 2**	**Model 3**	**Model 4**
	**OR (95% CI)**	**OR (95% CI)**	**OR (95% CI)**	**OR (95% CI)**
**Smoking social norms**				
**Family members who smoke**				
Father	0.79 (0.53–1.19)	0.79 (0.52–1.19)	0.84 (0.55–1.29)	0.83 (0.55–1.27)
Mother	1.45 (0.42–5.07)	1.35 (0.36–4.97)	1.44 (0.40–5.14)	1.41 (0.39–5.10)
Grandfather	1.35 (0.92–1.99)	1.35 (0.91–2.02)	1.40 (0.94–2.10)	1.40 (0.94–2.10)
Grandmother	0.83 (0.25–2.68)	0.84 (0.26–2.80)	0.82 (0.25–2.75)	0.79 (0.23–2.69)
**Parents' negative attitude**	0.39 (0.24–0.62)^***^	0.53 (0.32–0.87)^*^	0.64 (0.38–1.08)	0.64 (0.38–1.09)
**Number of family members who smoke**	1.06 (0.84–1.32)	1.06 (0.85–1.34)	1.01 (0.80–1.28)	1.01 (0.80–1.28)
**Teacher**				
School leadership	0.57 (0.32–1.01)	0.58 (0.32–1.05)	0.58 (0.32–1.05)	0.57 (0.31–1.05)
Male teachers	1.03 (0.64–1.65)	0.96 (0.58–1.58)	0.90 (0.54–1.49)	0.90 (0.54–1.49)
Female teachers	2.72 (1.27–5.83)^**^	2.85 (1.31–6.22)^**^	2.72 (1.24–5.97)^*^	2.65 (1.20–5.84)^*^
Head teachers	0.90 (0.39–2.08)	0.83 (0.35–1.98)	0.89 (0.37–2.12)	0.92 (0.38–2.18)
School support staff	1.46 (0.83–2.56)	1.24 (0.69–2.23)	1.21 (0.67–2.19)	1.24 (0.68–2.24)
**Peer**				
Classmates	1.26 (0.68–2.32)	1.22 (0.65–2.30)	1.27 (0.67–2.42)	1.28 (0.68–2.43)
Students in the same grade	1.30 (0.46–3.73)	1.40 (0.48–4.09)	1.36 (0.46–3.99)	1.34 (0.46–3.95)
Schoolmates	0.66 (0.22–1.98)	0.67 (0.22–2.04)	0.69 (0.23–2.11)	0.69 (0.23–2.11)
Friends	3.24 (1.85–5.68)^***^	2.56 (1.43–4.60)^**^	2.43 (1.35–4.37)^**^	2.44 (1.36–4.40)^**^
**Positive outcome expectation**				
A personal choice		1.29 (0.56–2.96)	1.01 (0.42–2.40)	0.99 (0.41–2.38)
A sign of maturity and success		0.82 (0.29–2.29)	0.78 (0.27–2.28)	0.80 (0.27–2.33)
Advantages greater than disadvantages		1.39 (0.54–3.56)	1.23 (0.46–3.30)	1.23 (0.46–3.32)
Cool and attractive		0.76 (0.34–1.71)	0.70 (0.31–1.60)	0.71 (0.31–1.62)
Social need		1.68 (1.01–2.80)^*^	1.46 (0.87–2.45)	1.45 (0.86–2.44)
Refresh and relieve boredom		2.86 (1.86–4.39)^***^	2.74 (1.77–4.23)^***^	2.74 (1.77–4.24)^***^
**Anti-smoking self-efficacy**				
Avoid smoking when someone smokes in front of you			0.66 (0.50–0.86)^**^	0.65 (0.49–0.86)^**^
Refusing a cigarette offered by others			0.62 (0.41–0.93)^*^	0.62 (0.42–0.93)^*^
Negative attitude to a stranger smoking in a no smoking area			1.18 (0.86–1.62)	1.18 (0.86–1.62)
**Attitudes toward control tobacco policies**				1.00 (0.77–1.30)

OR, odd ratios; 95% CI, 95% confidence intervals. ^*^p ≤ 0.05; ^**^p ≤ 0.01; ^***^p ≤ 0.001. All of the model control gender. There were 1,685 non-smokers and 210 who tried to smoke.

### 3.4. Multivariate logistic regression analyses of influencing factors of adolescents' smoking behaviors (current smoking vs. non-smoking)

Table 3 shows the results of multivariate logistic regression analyses of influencing factors of adolescents' smoking behaviors (current smoking vs. non-smoking). In Model 1, we analyzed the associations between social norms and adolescents' smoking behaviors. Parents' negative attitudes were a protective factor against current smoking (OR = 0.23, 95% CI: 0.11–0.45, *P* < 0.001). However, the number of family members smoking was a risk factor for current smoking (OR = 1.65, 95% CI: 1.08–2.51, *P* = 0.020), which means that the more family members who smoke, the more likely adolescents are to be current smokers. The adolescents who reported having female teachers and classmates who smoke were much more likely to be current smokers (OR = 3.53, 95% CI: 1.08–11.54, *P* = 0.037; OR = 4.26, 95% CI: 1.31–13.81, *P* = 0.016) than the adolescents who were not exposed to female teachers and classmates smoking. Based on Model 1, positive outcome expectations were added to Model 2. The adolescents who perceived that smoking is cool and attractive were more likely to be current smokers (OR = 9.64, 95% CI: 2.07–34.39, *P* < 0.001). We added anti-smoking self-efficacy variables in Model 3 based on Model 2. The adolescents who had more anti-smoking self-efficacy, such as skills avoiding smoking when someone smokes in front of them and refusing a cigarette offered by others, were less likely to smoke (OR = 0.53, 95% CI: 0.30–0.93, *P* = 0.028; OR = 0.20, 95% CI: 0.09–0.44, *P* < 0.001). Finally, we added the variable of attitudes toward control tobacco policies in Model 4 based on Model 3 and we found that it was a significant and protective predictor of current smoking behavior among adolescents (OR = 0.55, 95% CI: 0.32–0.95, *P* = 0.031).

**Table 3:** The influence of social norms, positive outcome expectations, anti-smoking self-efficacy and attitudes toward control tobacco policies on adolescents' smoking behavior (current smoking vs. non-smoking).

**Variable**	**Model 1**	**Model 2**	**Model 3**	**Model 4**
	**OR (95% CI)**	**OR (95% CI)**	**OR (95% CI)**	**OR (95% CI)**
**Smoking social norms**				
**Family members who smoke**				
Father	1.13 (0.46–2.78)	0.93 (0.33–2.62)	1.27 (0.40–4.06)	1.43 (0.43–4.74)
Mother	1.38 (0.22–8.85)	0.39 (0.04–3.51)	0.61 (0.05–7.45)	0.48 (0.03–6.91)
Grandfather	1.23 (0.52–2.92)	0.80 (0.27–2.35)	0.89 (0.27–2.91)	0.77 (0.23–2.59)
Grandmother	2.35 (0.50–11.00)	4.66 (0.87–24.82)	6.48 (0.95–44.31)	7.99 (1.16–55.06)^*^
**Parents' negative attitude**	0.23 (0.11–0.45)^***^	0.52 (0.23–1.22)	0.86 (0.33–2.27)	0.79 (0.30–2.07)
**Number of family members who smoke**	1.65 (1.08–2.51)^*^	1.99 (1.23–3.23)^**^	1.36 (0.76–2.43)	1.33 (0.73–2.42)
**Teacher**				
School leadership	3.15 (0.61–16.26)	3.20 (0.46–21.99)	2.53 (0.31–20.75)	3.26 (0.36–29.67)
Male teachers	0.28 (0.05–1.49)	0.25 (0.04–1.85)	0.27 (0.03–2.44)	0.20 (0.02–2.11)
Female teachers	3.53 (1.08–11.54)^*^	3.72 (0.95–14.50)	3.18 (0.76–13.31)	3.12 (0.74–13.08)
Head teachers	0.62 (0.15–2.53)	0.31 (0.06–1.53)	0.53 (0.10–2.73)	0.51 (0.10–2.71)
School support staff	3.72 (0.96–14.50)	3.93 (0.77–20.09)	4.32 (0.64–29.00)	4.44 (0.62–31.97)
**Peer**				
Classmates	4.26 (1.31–13.81)^*^	5.44 (1.34–22.12)^*^	4.99 (1.13–21.97)^*^	5.45 (1.16–25.49)^*^
Students in the same grade	0.17 (0.01–6.77)	0.19 (0.01–35.20)	0.54 (0.01–42.57)	0.43 (0.01–84.54)
Schoolmates	3.84 (0.10–151.75)	4.07 (0.02–719.56)	1.14 (0.02–83.78)	1.58 (0.01–298.49)
Friends	1.99 (0.68–5.85)	1.25 (0.33–4.78)	1.35 (0.34–5.46)	0.96 (0.21–4.36)
**Positive outcome expectation**				
A personal choice		1.06 (0.25–4.51)	0.63 (0.15–2.69)	0.62 (0.14–2.70)
A sign of maturity and success		0.82 (0.14–4.89)	0.62 (0.09–4.39)	0.86 (0.12–6.27)
Advantages greater than disadvantages		3.29 (0.65–16.76)	1.36 (0.22–8.60)	1.01 (0.16–6.44)
Cool and attractive		9.64 (2.70–34.39)^***^	7.67 (1.88–31.23)^**^	7.48 (1.80–31.02)^**^
Social need		2.60 (0.72–9.43)	1.94 (0.51–7.41)	2.18 (0.55–8.67)
Refresh and relieve boredom		2.13 (0.64–7.13)	1.34 (0.33–5.40)	1.14 (0.27–4.85)
**Anti-smoking self-efficacy**				
Avoid smoking when someone smokes in front of you			0.53 (0.30–0.93)^*^	0.67 (0.36–1.24)
Refusing a cigarette offered by others			0.20 (0.09–0.44)^***^	0.19 (0.08–0.43)^***^
Negative attitude to a stranger smoking in a no smoking area			1.07 (0.51–2.25)	1.15 (0.53–2.50)
**Attitudes toward control tobacco policies**				0.55 (0.32–0.95)^*^

OR, odd ratios; 95% CI, 95% confidence intervals. ^*^p ≤ 0.05; ^**^p ≤ 0.01; ^***^p ≤ 0.001. All of the model control gender.

## 4. Discussion

This study investigated the protective and risk factors for various smoking behaviors (current smoking and attempt at smoking) among school adolescents through SCT in Xiamen city, China. This study is promisor to provide evidence to understand the intra and interpersonal levels of psychosocial factors of adolescent smoking in a particular setting.

### 4.1. The influence of descriptive and injunctive social norms on adolescents' smoking behaviors

According to SCT, smoking behavior is determined by different social contexts (for example, peers, family, and school) providing adolescents with important role models (88). Several studies have discussed the social determinants of smoking among school adolescents (89–91). Adolescents have a greater prevalence of smoking behavior with parent smoking, teacher smoking, and peer smoking. Social cognitive learning occurs when an individual learns from other members of the group. Social-based decision-making may be driven by socio-cognitive activities related to social norms (92). Social norms and values possess adaptive properties that organize social cognition. A study of the factors influencing teenage smoking in India has found that smoking by parents and peers has an impact on teenage smoking behavior (93). The family, as children's first social group, affects the development of children's behaviors. Previous studies found that parental smoking increases the odds of an adolescent being a smoker (40). This study found that the number of smokers among adolescents' families showed no significant association with attempted smoking but did show a statistically significant association with current smoking. Parents' negative attitudes toward smoking reduce both the risk of exhibiting experimental smoking behavior and current smoking behaviors among adolescents. A previous study also found that parents' antismoking attitudes may help reduce the intention to start smoking among their children (94). Perceptions of smoking social norms related to family members influence adolescents' current smoking, while injunctive norms predict both the history of experimental smoking and engagement in current smoking. This study indicates that regions or municipalities should try to extend health education strategies and social norm interventions to correct the misperception of family norms to prevent adolescents' smoking behaviors.

Previous studies have analyzed the influence of descriptive and injunctive smoking social norms related to peers on health behaviors among adolescents (95). Peer influences are related more to descriptive norms than to injunctive norms. The current study discussed the associations between perceived smoking social norms related to peers and smoking behaviors among adolescents. The social norms mechanisms on smoking behavior included peer pressure and peer integration (96). When most of their peers around them smoke, adolescents are forced to smoke or reinforce their smoking behaviors to be accepted by the group due to peer pressure. Some scholars have also suggested that peer pressure to smoke is fundamentally a strategy to integrate with the group (97). Peer pressure is an important factor responsible for smoking initiation among adolescents. Adolescents are more likely to attempt to smoke when they see friends smoking, while current smoking is influenced by perceptions of classmates' behaviors. Intention to initiate smoking is significantly associated with the smoking status of friends and classmates among European adolescents (98). The inconsistent findings may be related to the quality and frequency of social interactions. In China, school life accounts for a large part of adolescents' lives, and adolescents spend much time with their classmates. Being around smoking peers frequently and feeling alienated from school places pressure on adolescents to transition from experimental smoking to current smoking. A study also found that experimental smokers are less likely than regular smokers to be surrounded by peers who smoke (99). Smoking interventions targeting adolescents at the experimentation stage are more effective than those at the habituation stage (100). Thus, adolescents who have friends with smoking behaviors should be targeted with interventions that halt the entrenchment of smoking. Peer education can effectively intervene in students' smoking behaviors (101), and school-based interventions should be developed to reduce the prevalence of current smoking and create a non-smoking social environment for school adolescents.

In the school environments, students' current smoking behavior tended to be related to perceived school members' smoking behaviors. The prevalence of smoking among adolescents in Fujian Province was 4.3%; while 47.2% of adolescents reported seeing someone smoking on campus, and 45.0% saw a teacher smoking (102). A study on 19 primary and middle schools in Xiamen city found that about 23.1% of students reported peer smoking (81). The difference between the actual prevalence of current smokers and perceived someone smoking behaviors in the school environment indicates that smoking intervention programs for adolescents should ask for more cooperation from school teachers, especially the supervising teachers, to monitor and restrict students' smoking behavior, as well as to limit their own smoking.

The Huli District of Xiamen takes adolescents as the key population of smoking prevention intervention and strives to reduce the number of new smokers. Some health education strategies have been implemented in the setting of this study (103). Public lectures on tobacco control were held in primary and secondary schools throughout the region to popularize the hazards of tobacco and tobacco control knowledge, and students' participation in a tobacco-free school propaganda work contest was held to create tobacco-free schools. However, health education programs in this study setting rarely include social norms on the mechanisms of interventions. Previous studies have indicated that smoking cessation interventions based on SCT are effective (104, 105). This study found that perceived smoking social norms related to family members, peers, and school female teachers could impact adolescents' smoking behaviors. This study indicates that regions or municipalities should try to extend health education strategies and social norm interventions to correct the misperception of smoking social norms to prevent adolescents' smoking behaviors.

The global prevalence of tobacco consumption among women is are still rising (106). Previous cultural norms in China have kept smoking among women at low levels, but China and the world are changing due to globalization and urbanization. With China's economic growth and cultural change along with the growing independence of women, smoking among Chinese women may also increase (107, 108). The prevalence of tobacco consumption among women is speedy increasing showing a change in social norms related to gender. Although this study controlled the gender of the participants, the social norms about female teacher smoking were related to adolescents' smoking behaviors. The norms against female smoking may be changing, and female smoking may be becoming more acceptable in China. It is important to monitor these norms and perceptions to prevent a rise in female smoking prevalence, and then reduce the prevalence of smoking behaviors among adolescents.

### 4.2. Smoking-positive outcome expectations influence smoking behaviors

SCT states that the outcome expectations, coupled with self-efficacy, determine the likelihood of engagement in a behavior (109). Positive reinforcement expectations are the expectations that individuals feel satisfaction from their behaviors, and they can permit individuals to proceed with the use of a substance. Positive outcome expectations are generally applied in theories of smoking motivation, which hold that smoking alleviates negative affect (110). This study combined with previous studies indicates that positive smoking expectations are significantly correlated with smoking behavior (63, 111, 112). The beliefs that “smoking is a social demand” and “smoking can refresh and relieve boredom” are associated with experimental smoking, while the belief that “smoking is cool and makes you more attractive” appears to predict current smoking. The perception that “smoking is cool and makes you more attractive” may be related to the marketing strategies targeted at adolescents. Messages and fake images that reflect the qualities teenagers value, such as beauty, coolness, popularity, dependence, sexiness, and being attractive, are used by tobacco companies for their brands to attract the general public, especially the youth population (113, 114). Middle school students are at a special stage of psychological development. These advertisements may shift adolescents' attitudes and beliefs. Young people may easily be affected by a bad social atmosphere and have negative attitudes or incorrect values toward smoking behaviors. The perception of smoking behavior as making one cooler and symbolizing masculinity is statistically significant for smoking behavior (115). The empirical literature suggests that tobacco advertising bans do play an important role in reducing tobacco consumption in developing countries (116). This study would call for the expansion and implementation of the band of tobacco advertising to decrease the smoking-positive outcome expectations that influence smoking behavior among China's adolescents.

The other two ideas, “smoking is a social demand” and “smoking can refresh and relieve boredom,” are related to the social need to belong and individual needs. SCT describes an interaction between person, environment, and behavior. Individuals may examine the likely implications of alternative actions and evaluate the anticipated consequences (117). Motivated behavior arises through the expectation of reward or avoidance of punishment. The need to belong and immediate social gain are major themes influencing adolescents smoking decisions (118). Around campus, at home, and in cyberspace, tobacco is always tempting young people. If adolescents perceive that society and their peers approve of smoking, they would smoke to meet the need for belonging and social integration (44). Thus, we should always pay attention to the changes in middle school students' emotions and personalities, relieve their negative emotions related to pressure and anxiety promptly, and reduce their motivation to smoke. This study suggests that health education workers can reduce the rewarding effects of smoking, such as social demand, individual needs, and incorrect minds. Family, schools, and social media who want to prevent adolescents from smoking might consider focusing their efforts on establishing a good quality of communication on smoking harmful effects without discussion of smoking-positive outcome expectations. Future studies should analyze the effect of employing expectancy challenge strategies and cognitive restructuring interventions on reducing smoking behavior among adolescents.

### 4.3. The role of anti-smoking self-efficacy in preventing experimental and current smoking

Anti-smoking self-efficacy is the ability of adolescents to not smoke and abstain from smoking in high-risk situations (64). Self-efficacy affects not only students' attitudes toward smoking control, but also their smoking behaviors. Adolescents with low self-efficacy have a 5–17 times higher risk of smoking than those with high self-efficacy (119). This study also found that anti-smoking self-efficacy is a protective factor against smoking behaviors among adolescents. A self-efficacy promotion program can be recommended for smoking cessation in adolescents (120). A high level of self-efficacy enables individuals to make better use of cognitive resources to establish healthy behaviors to resist temptation. Health education should be developed for middle school students by improving the knowledge system of smoking prevention, enhancing their cognitive levels, and improving their anti-smoking self-efficacy. Our findings indicate that refusing cigarettes offered by others and developing self-discipline to avoid smoking when someone smokes in front of you were negatively associated with smoking behaviors. In China's social culture, “social smoking” is an important factor influencing smoking behavior. A high level of self-efficacy plays a very important role in resisting external factors such as peer influence, advertising and social atmosphere (121). Disseminating information about the health risks of smoking and teaching adolescents to say no are important strategies for preventing smoking.

### 4.4. Adolescents' favorable attitudes toward tobacco control policies help create non-smoking campuses

Tobacco use remains the leading cause of preventable mortality, disability, and death. Especially in some developing countries with similar characteristics to China, smoking is an important cause of the occurrence and death of non-communicable diseases. Smoking kills about a million people in India, 10 percent of all deaths (122), which is also happening in Africa. The overall prevalence of smoking among school children in East Africa is currently 9.02 percent and the prevalence of smoking is increasing year by year (123). Smoke-free policies have been implemented as a public health measure to reduce smoking among adolescents (124). To protect middle school students from the harm of tobacco and reduce the smoking rates of adolescents, China has issued a series of policies and regulations from the source, the environment, and other aspects, such as opinions on further strengthening tobacco control in schools (125), reducing the smoking rate of people over 15 years old to 20% by 2030 (126), guidelines for building smoke-free schools and the requirement to post smoke-free signs (127). In 2021, the proportion of middle school students trying to smoke cigarettes and smoking cigarettes was 16.7 and 4.7%, respectively, which decreased by 1.2 percentage points compared with 2019, indicating that the implementation of policies and regulations has obtained certain success (128). These policies also have certain reference significance for some developing countries such as India and Africa. The existing research literature indicated that favorable attitudes toward tobacco control policies can contribute to their effective implementation and success in changing tobacco-related attitudes and behaviors (129–131). This study found that adolescents' attitudes toward control tobacco policies can predict the likelihood of current smoking behavior. To implement a tobacco-free campus policy, we suggest educational campaigns that focus on adolescent support for tobacco control policies.

Country control tobacco policies are very relevant from a global perspective. Previous studies have indicated that country-control tobacco policies are effective in reducing smoking prevalence (132). Some scholars predicted that if the MPOWER package policy had been implemented globally starting in 2010 with a 100% price increase for cigarettes, global cigarette smoking prevalence would be 13.2% in 2030 (523 million smokers) (133). School tobacco control policies are associated with reduced odds of smoking initiation among youth (134), and A combination of several strategies is likely to be most effective in decreasing smoking rates (135). Research reported that comprehensive tobacco control programs lead to an 8% (4–12%) over a 5-year time horizon relative reduction, increasing to a 12% (6–18%) over a 40-year time horizon relative reduction in smoking prevalence through the greater impact on youth smoking (136). To further improve the implementation effect of the anti-smoking policy, the smoke-free public settings for young people and the acceptability of smoking policies and tobacco control measures among adolescents should be increased.

## 5. Strengths and limitations

Although several previous studies have explored the influencing factors of smoking behaviors among adolescents, this study assessed and compared the relationships of social norms, positive outcome expectations, anti-smoking self-efficacy, and attitudes toward control tobacco policies with smoking behaviors (such as experimental smoking and current smoking). This study analyzed the associations of several intra- and interpersonal levels of psychosocial factors on different smoking behaviors among adolescents. Several studies have discussed the associations between peers' and parents' norms on adolescents' smoking behaviors (137, 138). Our model included social norms within adolescents' families, peers, and school settings simultaneously to avoid omitted variable bias. Female teachers smoking, friends smoking and positive outcome expectation (the beliefs that “smoking is a social demand” and “smoking can refresh and relieve boredom”) were risk factors of adolescents' experimental smoking behavior. Parents' negative attitudes and individual anti-smoking self-efficacy (such as avoiding smoking when someone smokes in front of them and refusing a cigarette offered by others) were protective factors of adolescents' experimental smoking behavior. Reducing exposure to female teachers and friends smoking, and parental or supervisory monitoring to develop correct cognition of smoking among adolescents were considered suitable means of reducing adolescents' experimental smoking. This study also emerged that reductions in adolescents' exposure to family, female teachers, and classmates smoking situations, reductions in positive outcome expectation (the belief that smoking is cool and attractive), improving anti-smoking self-efficacy, and favorable attitudes toward control tobacco policies were considered of value in terms of being capable of reducing adolescents' current smoking. The government in China has introduced a notice on further strengthening tobacco control work for adolescents. This tobacco control policy emphasizes establishing and improving long-term mechanisms for adolescences tobacco control, including tobacco control advocates and guidance, advocating for adolescents to refuse the first cigarette, and the construction of smoke-free schools. The findings of this study are critical to helping the local implementation of smoke-free campus policies.

This study has some limitations. First, this study used a cross-sectional design, and as a result, the causal relationship between smoking behavior and its related factors needs to be further explored. Second, adolescents' different smoking behavior statuses, including never smoking, experimental smoking, and current smoking, were measured by self-reported questions, which increases measurement errors. However, self-reported smoking behavior is generally considered a valid measure (139). Measurement errors are unlikely to be a serious concern. Third, due to the limitation of the questionnaire length, the survey items of this study could not cover all possible influencing factors of adolescent smoking behavior, such as negative outcome expectations or risk perception of smoking behavior. The negative outcome expectations of smoking do not promote teenagers' smoking behavior. Once teenagers are aware of negative outcome expectations or correct risk perception (such as harmful to health, damaging teeth, etc.), they are less likely to smoke. Future studies could include this in the survey. In addition, the dependent variables of smoking behaviors for the regression analysis did not include vaping or e-cigarette use, which could cause biased results and be a limitation of this study, due to the current global demand for information about novel tobacco products among adolescents. Finally, data were derived from four middle schools in a city in China. Due to different regions and differences in cultural cognition, these schools have demographic and other characteristics that make them differ from other schools in China. Thus, this study cannot be generalized to the population of school adolescents in China. However, this study took individual, social, and policy factors into consideration to propose appropriate strategies for reducing experimental smoking and current smoking, which could have some theoretical and practical implications for health education professionals, society, and policy-makers.

## 6. Conclusion

In summary, this study explored various models of smoking behavior among Chinese adolescents and found that social norms, positive outcome expectations, anti-smoking self-efficacy, and attitudes toward control tobacco policies influenced adolescents' experimental smoking and current smoking behaviors to varying degrees. This suggests that the above-influencing factors should be fully addressed by prevention and intervention programs for adolescent smoking. A multipronged approach should be used to create smoke-free families, smoke-free schools, and even a smoke-free society by combining the efforts of family, school, society, and government to keep the majority of adolescents away from tobacco.

## Data availability statement

The datasets generated for this study are available on request to the corresponding author.

## Ethics statement

Ethical approval was not provided for this study on human participants because the data used was derived from a 2017 cross-sectional survey of cigarette smoking among junior Chinese middle school students in Xiamen. This was part of a Chinese middle school students tobacco survey, named “Notice of Xiamen Municipal Health and Family Planning Commission on printing and distributing the Implementation Plan of Xiamen Residents' Health Literacy Promotion Project in 2017”. This was conducted by the Xiamen Municipal Health and Family Planning Commission. The study did not receive Ethical Committee approval. This study followed the guidelines issued in the Declaration of Helsinki where applicable. Written informed consent to participate in this study was provided by the participants' legal guardian/next of kin.

## Author contributions

Y-CC has full access to all of the data in the study and take responsibility for the integrity of the data and the accuracy of the data analysis. Y-CC, MC, ML, and XL conceived of the study, participated in its design and coordination, and drafted the manuscript. ML performed the data acquisition and sampling. HM, ZF, LM, and PW contributed to interpretation, discussion of result, and writing and critical revision of the article. TC made the acquisition of data and critical review of the manuscript for important intellectual content. All authors approved the final version and all take responsibility for its content.

## References

[1] Chen X Li Y Unger JB Gong J Johnson CA Guo Q. Hazard of smoking initiation by age among adolescents in Wuhan, China. Prev Med. (2001) 32:437–45. 10.1006/pmed.2001.0826 11330994

[2] Ma H Unger JB Chou CP Sun P Palmer PH Zhou Y. Risk factors for adolescent smoking in urban and rural China: findings from the China seven cities study. Addict Behav. (2008) 33:1081–5. 10.1016/j.addbeh.2008.04.004 18495363

[3] Sheng Xiong P Juan Xiong M Xi Liu Z Liu Y. Prevalence of smoking among adolescents in China: an updated systematic review and meta-analysis. Public Health. (2020) 182:26–31. 10.1016/j.puhe.2020.01.011 32145409

[4] Yang X Shi F He Y Zhu J. Analysis of the effects of second-hand smoke exposure on smoking behavior and future smoking intention of adolescents. In: The 20th National Tobacco Control Symposium of Chinese Association on Tobacco Control, and the 10th Cross-Strait, Hong Kong and Macau Tobacco Prevention and Control Symposium. Chongqing (2019).

[5] Zuo J. The effect of smoking environment on smoking behavior in adolescents: a moderated mediating model (Master's thesis). Hunan Normal University, Changsha, China. (2021).

[6] Shuhui Zhang HZ Haodong G. The impact of social determinants of health on addictive behavior in adolescents. Chinese Youth Soc Sci. (2018) 37:83–91. 10.1016/S0140-6736(12)60149-4 34653928

[7] Yang Y. Does economic growth induce smoking?—evidence from China. Emp Econ. (2022) 63:821–45. 10.1007/s00181-021-02155-8

[8] Chiang Y-C Gao D-R Li X Lee C-Y Sun X-Y Chen C-T. Effect of urban transformation and the age-friendly city strategy on the improvement of residents' subjective well-being: simulation based on the scenario method. J Urban Plan Develop. (2021) 147:04021044. 10.1061/(ASCE)UP.1943-5444.000074029515898

[9] Xiamen Bureau of Statistics. Xiamen 2021 National Economic and Social Development Statistics Bulletin. (2022). Available online at: http://tjj.xm.gov.cn/tjzl/ndgb/202203/t20220322_2636525.htm (accessed January 2, 2023).

[10] Cheng TO. Adolescent smoking in China. Int J Cardiol. (2008) 126:1–2. 10.1016/j.ijcard.2007.04.168 17904665

[11] Qiong-Wang YW Qiyun D Xiaolu F Yong J Wenjing Z Jiang L. Prevalence of smoking among adolescents in Chengdu. Mod Pre Med. (2005) 32:1139–40.

[12] Xiaosheng X Jianjun L. Study on tobacco control effect of primary and middle school students in Changsha City. Chin Prev Med. (2005) 6:289–300.

[13] Mingxia Y Chengmeng T Qiang Z Shi-min L Sha-sha S Jiayi C. Study on the influencing factors of new smoking behavior among rural middle school students in Zizhong County, Sichuan Province. Mod Prev Med. (2021) 48:2797–801.

[14] Beibei Che JG Chen D Xiaoxian J Kun X Jian W Chenchen X Jinming Y. A case-control study of the effect of e-cigarette experimentation on smoking propensity among adolescents.Chin J School Health. (2020) 41:1657–60. 10.16835/j.cnki.1000-9817.2020.11.01631958678

[15] Soneji S Barrington-Trimis JL Wills TA Leventhal AM Unger JB Gibson LA. Association between initial use of e-cigarettes and subsequent cigarette smokingamongadolescents and young adults a systematic review and meta-analysis. JAMA Pediatr. (2017) 171:788–97. 10.1001/jamapediatrics.2017.1488 28654986 PMC5656237

[16] Perry CL Baranowski T Parcel GS. How individuals, environments, and health behavior interact: social cognitive theory. In:Glanz K Lewis FM Rimer BK, editors. Health Behavior and Health Education, Theory Research and Practice. San Francisco: Jossey-Bass, Inc (1997). p. 153–78.

[17] Bandura A. Social cognitive theory of mass communication. Media Psychol. (2001) 3:265–99. 10.1207/S1532785XMEP0303_03

[18] Self-efficacy. Social Foundations of Thought and Action: A Social Cognitive Theory. Englewood Cliffs, NJ: Prentice-Hall (1986). p. 617.

[19] Kilford EJ Garrett E Blakemore SJ. The development of social cognition in adolescence: an integrated perspective. Neurosci Biobehav Rev. (2016) 70:106–20. 10.1016/j.neubiorev.2016.08.016 27545755

[20] Caskey MM Anfara VA. Research Summary: Young Adolescents' Developmental Characteristics. (2007). Available online at: http://www.nmsa.org/Research/ResearchSummaries/DevelopmentalCharacteristics/tabid/1414/Default.aspx (accessed January 3, 2023).

[21] Bagherniya M Sharma M Mostafavi F Keshavarz SA. Application of social cognitive theory in predicting childhood obesity prevention behaviors in overweight and obese Iranian adolescents. Int Q Community Health Educ. (2015) 35:133–47. 10.1177/0272684X15569487 25856805

[22] Tekin U Erermis HS Satar A Aydin AN Kose S Bildik T. Social cognition in first episode adolescent depression and its correlation with clinical features and quality of life. Clin Child Psychol Psychiatry. (2021) 26:140–53. 10.1177/1359104520973254 33246372

[23] Cervantes CM Porretta DL. Impact of after school programming on physical activity among adolescents with visual impairments. Adapt Phys Act Quarterly. (2013) 30:127–46. 10.1123/apaq.30.2.127 23520243

[24] Anderson-Bill ES Winett RA Wojcik JR. Social cognitive determinants of nutrition and physical activity among web-health users enrolling in an online intervention: the influence of social support, self-efficacy, outcome expectations, and self-regulation. J Med Internet Res. (2011) 13:e28. 21441100 10.2196/jmir.1551 PMC3221350

[25] Michie S Abraham C. Interventions to change health behaviours: evidence-based or evidence- inspired? Psychol Health. (2004) 19:29–49. 10.1080/0887044031000141199

[26] Noar SM Chabot M Zimmerman RS. Applying health behavior theory to multiple behavior change: considera tions and approaches. Prev Med. (2008) 46:275–80. 10.1016/j.ypmed.2007.08.001 17825898

[27] Social Foundations of Thought and Action: A Social Cognitive Theory. Englewood Cliffs, NJ: Prentice-Hall (1986).

[28] McMillan B Higgins AR Conner M. Using an extended theory of planned behaviour to understand smoking amongst schoolchildren. Addict Res Theory. (2005) 13:293–306. 10.1080/16066350500053679

[29] ter Doest L Dijkstra A Gebhardt WA Vitale S. Cognitions about smoking and not smoking in adolescence. Health Educ Behav. (2009) 36:660–72. 10.1177/1090198107301329 17620668

[30] Harakeh Z Scholte RH Vermulst AA de Vries H Engels RC. Parental factors and adolescents' smoking behavior: an extension of the theory of planned behavior. Prev Med. (2004) 39:951–61. 10.1016/j.ypmed.2004.03.036 15475029

[31] Jiménez TI Estévez E. School aggression in adolescence: Examining the role of individual, family and school variables. Int J Clin Health Psychol. (2017) 17:251–60. 10.1016/j.ijchp.2017.07.002 30487900 PMC6220901

[32] Bauman KE Ennett ST. Peer influence on adolescent drug-use–comment. Am Psychol. (1994) 49:820–2. 10.1037/0003-066X.49.9.820 7978669

[33] Geckova AM Stewart R van Dijk JP Orosova O Groothoff JW Post D. Influence of socio-economic status, parents and peers on smoking behaviour of adolescents. Eur Addict Res. (2005) 11:204–9. 10.1159/000086403 16110228

[34] Cook EC Buehler C Henson R. Parents and peers as social influences to deter antisocial behavior. J Youth Adolesc. (2009) 38:1240–52. 10.1007/s10964-008-9348-x 19669903

[35] Page RM Nguyen Thanh H Hoang Khanh C Truong Quang T. Social normative beliefs about smoking among vietnamese adolescents. Asia-Pacific J Public Health. (2012) 24:68–81. 10.1177/1010539510370993 20498122

[36] Manuel Andreu J Elena Pena M Larroy C. Antisocial behavior, impulsiveness and justification beliefs: analysis of their interrelationships with proactive and reactive aggression in adolescents. Behav Psychol Psicologia Conductual. (2010) 18:57–72.

[37] Turner JC. Social Influence. Belmont, CA, US: Thomson Brooks/Cole Publishing Co (1991). xvi, 206–xvi, p.

[38] Higgs S Thomas J. Social influences on eating. Curr Opin Behav Sci. (2016) 9:1–6. 10.1016/j.cobeha.2015.10.005

[39] Borsari B Carey KB. Descriptive and injunctive norms in college drinking: a meta-analytic integration. J Stud Alcohol. (2003) 64:331–41. 10.15288/jsa.2003.64.331 12817821 PMC2431131

[40] Chen XG Stanton B Fang XY Li XM Lin DH Zhang JT. Perceived smoking norms, socioenvironmental factors, personal attitudes and adolescent smoking in China: a mediation analysis with longitudinal data. J Adolescent Health. (2006) 38:359–68. 10.1016/j.jadohealth.2005.03.010 16549296

[41] LaMorte WW. Behavioral Change Models. (2019). Available online at: https://sphweb.bumc.bu.edu/otlt/mph-modules/sb/behavioralchangetheories/behavioralchangetheories5.html (accessed November 7, 2022).

[42] Kotchick BA Shaffer A Forehand R. Adolescent sexual risk behavior: a multi-system perspective. Clin Psychol Rev. (2001) 21:493–519. 10.1016/S0272-7358(99)00070-7 11413865

[43] Donovan JE. Adolescent alcohol initiation: a review of psychosocial risk factors. J Adoles Health. (2004) 35:529.e7–18. 10.1016/j.jadohealth.2004.02.003 15581536

[44] East K McNeill A Thrasher JF Hitchman SC. Social norms as a predictor of smoking uptake among youth: a systematic review, meta-analysis and meta-regression of prospective cohort studies. Addiction. (2021) 116:2953–67. 10.1111/add.15427 33538370

[45] Eisenberg ME Forster JL. Adolescent smoking behavior: measures of social norms. Am J Prev Med. (2003) 25:122–8. 10.1016/S0749-3797(03)00116-8 12880879

[46] Kong G Camenga D Cavallo D Connell CM Pflieger JC Krishnan-Sarin S. The role of ethnic pride and parental disapproval of smoking on smoking behaviors among minority and white adolescents in a suburban high school. Am J Addict. (2012) 21:424–34. 10.1111/j.1521-0391.2012.00266.x 22882393 PMC3422755

[47] Ahern J Galea S Hubbard A Syme SL. Neighborhood smoking norms modify the relation between collective efficacy and smoking behavior. Drug Alcohol Depend. (2009) 100:138–45. 10.1016/j.drugalcdep.2008.09.012 19010610 PMC2664307

[48] Gorukanti A Delucchi K Ling P Fisher-Travis R Halpern-Felsher B. Adolescents' attitudes towards e-cigarette ingredients, safety, addictive properties, social norms, and regulation. Prev Med. (2017) 94:65–71. 10.1016/j.ypmed.2016.10.019 27773711 PMC5373091

[49] Hagman BT Clifford PR Noel NE. Social norms theory-based interventions: testing the feasibility of a purported mechanism of action. J Am College Health. (2007) 56:293–8. 10.3200/JACH.56.3.293-298 18089512

[50] Latkin C Donnell D Liu T-Y Davey-Rothwell M Celentano D Metzger D. The dynamic relationship between social norms and behaviors: the results of an HIV prevention network intervention for injection drug users. Addiction. (2013) 108:934–43. 10.1111/add.12095 23362861 PMC3628934

[51] Flynn BS Worden JK Bunn JY Solomon LJ Ashikaga T Connolly SW. Mass media interventions to reduce youth smoking prevalence. Am J Prev Med. (2010) 39:53–62. 10.1016/j.amepre.2010.03.008 20537841 PMC2898197

[52] Feather NT. Values, valences, expectations, and actions. J Soc Issues. (1992) 48:109–24. 10.1111/j.1540-4560.1992.tb00887.x

[53] Feather NT. Subjective probability and decision under uncertainty. Psychol Rev. (1959) 66:150–64. 10.1037/h0045692 13658302

[54] Smit K Voogt C Hiemstra M Kleinjan M Otten R Kuntsche E. Development of alcohol expectancies and early alcohol use in children and adolescents: a systematic review. Clin Psychol Rev. (2018) 60:136–46. 10.1016/j.cpr.2018.02.002 29449029

[55] Pinquart M Borgolte K. Change in alcohol outcome expectancies from childhood to emerging adulthood: a meta-analysis of longitudinal studies. Drug Alcohol Rev. (2022) 41:1216–25. 10.1111/dar.13454 35238083

[56] Spruijt-Metz D Gallaher P Unger JB Johnson CA. Unique contributions of meanings of smoking and outcome expectancies to understanding smoking initiation in middle school. Ann Behav Med. (2005) 30:104–11. 10.1207/s15324796abm3002_2 16173906

[57] Wahl SK Turner LR Mermelstein RJ Flay BR. Adolescents' smoking expectancies: Psychometric properties and prediction of behavior change. Nicot Tobacco Res. (2005) 7:613–23. 10.1080/14622200500185579 16085531

[58] Josendal O Aaro LE. Adolescent smoking behavior and outcome expectancies. Scand J Psychol. (2012) 53:129–35. 10.1111/j.1467-9450.2011.00927.x 22150552

[59] Shadur JM Ninnemann AL Lim A Lejuez CW MacPherson L. The prospective relationship between distress tolerance and cigarette smoking expectancies in adolescence. Psychol Add Behav. (2017) 31:625–35. 10.1037/adb0000300 28714727

[60] Carels RA Darby LA Rydin S Douglass OM Cacciapaglia HM O'Brien WH. The relationship between self-monitoring, outcome expectancies, difficulties with eating and exercise, and physical activity and weight loss treatment outcomes. Ann Behav Med. (2005) 30:182–90. 10.1207/s15324796abm3003_2 16336069

[61] Baker TB Brandon TH Chassin L. Motivational influences on cigarette smoking. Annu Rev Psychol. (2004) 55:463–91. 10.1146/annurev.psych.55.090902.142054 14744223

[62] Austin SB Gortmaker SL. Dieting and smoking initiation in early adolescent girls and boys: a prospective study. Am J Public Health. (2001) 91:446–50. 10.2105/AJPH.91.3.446 11236412 PMC1446587

[63] Urban R. Smoking outcome expectancies mediate the association between sensation seeking, peer smoking, and smoking among young adolescents. Nicotine Tobacco Res. (2010) 12:59–68. 10.1093/ntr/ntp174 19959571 PMC2802571

[64] Hiemstra M Otten R de Leeuw RNH van Schayck OCP Engels R. The changing role of self-efficacy in adolescent smoking initiation. J Adolescent Health. (2011) 48:597–603. 10.1016/j.jadohealth.2010.09.011 21575820

[65] Hiemstra M Otten R Engels RC. Smoking onset and the time-varying effects of self-efficacy, environmental smoking, and smoking-specific parenting by using discrete-time survival analysis. J Behav Med. (2012) 35:240–51. 10.1007/s10865-011-9355-3 21643802 PMC3305880

[66] Bektas M Ozturk C Armstrong M. An approach to children's smoking behaviors using social cognitive learning theory. Asian Pacific J Canc Prevention. (2010) 11:1143–9. 21133639

[67] Shadel WG Niaura R Goldstein MG Abrams DB. Cognitive avoidance as a method of coping with a provocative smoking cue: the moderating effect of nicotine dependence. J Behav Med. (2001) 24:169–82. 10.1023/A:1010762631464 11392918

[68] Chang FC Lee CM Lai HR Chiang JT Lee PH Chen WJ. Social influences and self-efficacy as predictors of youth smoking initiation and cessation: a 3-year longitudinal study of vocational high school students in Taiwan. Addiction. (2006) 101:1645–55. 10.1111/j.1360-0443.2006.01607.x 17034445

[69] Shadel WG Cervone D. Evaluating social-cognitive mechanisms that regulate self-efficacy in response to provocative smoking cues: an experimental investigation. Psychol Add Behav. (2006) 20:91–6. 10.1037/0893-164X.20.1.91 16536671

[70] Fagan P Eisenberg M Frazier L Stoddard AM Avrunin JS Sorensen G. Employed adolescents and beliefs about self-efficacy to avoid smoking. Addict Behav. (2003) 28:613–26. 10.1016/S0306-4603(02)00227-7 12726779

[71] Kinard BR Webster C. The effects of advertising, social influences, and self-efficacy on adolescent tobacco use and alcohol consumption. J Consum Affairs. (2010) 44:24–43. 10.1111/j.1745-6606.2010.01156.x

[72] Shadel WG Fryer CS Tharp-Taylor S. Uncovering the most effective active ingredients of antismoking public service announcements: the role of actor and message characteristics. Nicotine Tobacco Res. (2009) 11:547–52. 10.1093/ntr/ntp045 19372574 PMC2671467

[73] Setodji CM Martino SC Scharf DM Shadel WG. Friends moderate the effects of pro-smoking media on college students' intentions to smoke. Psychol Addict Behav. (2013) 27:256–61. 10.1037/a0028895 22686961 PMC3637912

[74] Nyman J Parisod H Axelin A Salantera S. Finnish adolescents' self-efficacy in peer interactions: a critical incident study. Health Promot Int. (2019) 34:961–9. 10.1093/heapro/day048 30020443

[75] Buchmann AF Blomeyer D Jennen-Steinmetz C Schmidt MH Esser G Banaschewski T. Early smoking onset may promise initial pleasurable sensations and later addiction. Addict Biol. (2013) 18:947–54. 10.1111/j.1369-1600.2011.00377.x 21966958

[76] Obieche O Lee M Salehi N. Exploring attitudes towards smoking behaviour and cessation among hospitalised smokers via a socio-ecological framework: a scoping review. Add Behav. (2021) 122:107040. 10.1016/j.addbeh.2021.107040 34246988

[77] Janssen E Le Nezet O Shah J Chyderiotis S Brissot A Philippon A. Increasing socioeconomic disparities in tobacco smoking decline among French adolescents (2000-2017). J Public Health. (2020) 42:E449–E57. 10.1093/pubmed/fdz135 31774505

[78] Sanders LM Shaw JS Guez G Baur C Rudd R. Health literacy and child health promotion: implications for research, clinical care, and public policy. Pediatrics. (2009) 124(Suppl. 3):S306–14. 10.1542/peds.2009-1162G 19861485

[79] Tang JJ Phoenix KH. The new smoking ban policy in Hong Kong: relationship between attitudes toward the policy and social cognitive determinants of smoking. Int J Psychol. (2008) 43:64.

[80] Youlan Chen LD Ding T Biao R Hong Y Wenjian W. Evaluation on the effect of tobacco control intervention in primary and secondary schools in Xiamen. Chin J School Health. (2014) 35:425–8. 10.16835/j.cnki.1000-9817.2014.03.042

[81] Youlan Chen LD Ding T Biao R Hong Y Wenjian W. Analysis on the current situation and influencing factors of primary and secondary school students' attempt to smoke in Xiamen. Chin J School Health. (2014) 35:886–9. 10.16835/j.cnki.1000-9817.2014.06.031

[82] Group GYTSC. Global Youth Tobacco Survey (GYTS): Core Questionnaire with Optional Questions, Version 1.2. Atlanta, GA: Centers for Disease Control and Prevention (2014).

[83] Zhang L Wang W Zhao Q Vartiainen E. Psychosocial predictors of smoking among secondary school students in Henan, China. Health Educ Res. (2000) 15:415–22. 10.1093/her/15.4.415 11066459

[84] Wen X Chen W Liang C. Effect of health promotion school model on smoking prevention and control in secondary school students. Chin J Public Health. (2007) 23:782–4.

[85] World Health Organization. Guidelines for Controlling and Monitoring the Tobacco Epidemic. (1998). Available online at: https://apps.who.int/iris/handle/10665/42049 (accessed October 20, 2022).

[86] Lamorte WW. Social Norms Theory. (2019). Available online at: https://sphweb.bumc.bu.edu/otlt/MPH-Modules/SB/BehavioralChangeTheories/BehavioralChangeTheories7.html (accessed October 25, 2022).

[87] Self-efficacy. Social Foundations of Thought and Action: A Social Cognitive Theory. Englewood Cliffs, NJ: Prentice-Hall (1986). p. 390–453.

[88] Piontek D Buehler A Rudolph U Metz K Kroeger C Gradl S. Social contexts in adolescent smoking: does school policy matter? Health Educ Res. (2008) 23:1029–38. 10.1093/her/cym063 17947247

[89] Geckova A van Dijk JP van Ittersum-Gritter T Groothoff JW Post D. Determinants of adolescents' smoking behaviour: a literature review. Cent Eur J Public Health. (2002) 10:79–87. 12298346

[90] Cheng X Guo X Jin C. Social determinants of smoking among school adolescents in Beijing, China. Tob Induc Dis. (2022) 20:73. 10.18332/tid/152202 36118554 PMC9426650

[91] Do EK Bradley KC Fugate-Laus K Kaur K Halquist MS Ray L. An examination of social and environmental determinants of secondhand smoke exposure among non-smoking adolescents. Tobacco Preven Cessation. (2021) 7:20. 10.18332/tpc/131875 33728387 PMC7954078

[92] Zinchenko O Arsalidou M. Brain responses to social norms: meta-analyses of fMRI studies. Hum Brain Mapp. (2018) 39:955–70. 10.1002/hbm.23895 29160930 PMC6866581

[93] Arora V Gupta N Gupta P Bansal M Thakar S Nagpal I. Cigarette smoking behavior and associated psychosocial determinants among school going adolescents in Panchkula, India. J Indian Assoc Public Health Dentistry. (2017) 15:27–31. 10.4103/2319-5932.201944

[94] Chassin L Presson CC Sherman SJ Edwards DA. The natural-history of cigarette-smoking. - predicting young-adult smoking outcomes from adolescent smoking patterns. Health Psychol. (1990) 9:701–16. 10.1037/0278-6133.9.6.701 2286181

[95] Baumgartner SE Valkenburg PM Peter J. The influence of descriptive and injunctive peer norms on adolescents' risky sexual online behavior. Cyberpsychol Behav Soc Network. (2011) 14:753–8. 10.1089/cyber.2010.0510 22017408

[96] Stjerna M-L Lauritzen SO Tillgren P. “Social thinking” and cultural images: teenagers' notions of tobacco use. Soc Sci Med. (2004) 59:573–83. 10.1016/j.socscimed.2003.11.003 15144766

[97] Lakon CM Valente TW. Social integration in friendship networks: the synergy of network structure and peer influence in relation to cigarette smoking among high risk adolescents. Soc Sci Med. (2012) 74:1407–17. 10.1016/j.socscimed.2012.01.011 22436575 PMC3736845

[98] Masood M Masood Y Sabri BAM Younis LT Yusof N Reidpath D. Within-family discussion on harmful effects of smoking and intention to initiate smoking among european adolescents. J Addict Med. (2015) 9:261–5. 10.1097/ADM.0000000000000127 26241085

[99] Ausems M Mesters I Van Breukelen G De Vries H. Do dutch 11-12 years olds who never smoke, smoke experimentally or smoke regularly have different demographic backgrounds and perceptions of smoking? Eur J Public Health. (2003) 13:160–7. 10.1093/eurpub/13.2.160 12807093

[100] Harken LS. The prevention of adolescent smoking:a public health priority. Eval Health Prof. (1987) 10:373–93. 10.1177/016327878701000402

[101] Dobbie F Purves R McKell J Dougall N Campbell R White J. Implementation of a peer-led school based smoking prevention programme: a mixed methods process evaluation. BMC Public Health. (2019) 19:742. 10.1186/s12889-019-7112-7 31196124 PMC6567418

[102] The Health Commission of Fujian Province. The latest Survey Shows That the Province's Youth Now Smoking Rate was 4.3% in 2019. (2020). Available online at: https://wjw.fujian.gov.cn/jggk/csxx/ghyxxc/fzgh_41281/202012/t20201203_5471398.htm (accessed January 9, 2023).

[103] Huli District People's Government of Xiamen City. Huli District People's Government Office on the Issuance of Huli District to Further Strengthen the Implementation of the New Period of Patriotic Health Work Notice. (2016). Available online at: http://www.huli.gov.cn/zwgk/zfxxgkzl/bmzfxxgk/zfb/zfxxgkml/ghjh/201710/t20171018_102206.htm

[104] Zheng P Zheng L Guo F Xiao X. [Evaluation of two-year follow-up of group intervention on smoking cessation based on social cognitive theory]. J. Hyg. Res. (2008) 37:53–6. 18421865

[105] Villanti AC West JC Klemperer EM Graham AL Mays D Mermelstein RJ. Smoking-cessation interventions for US young adults: updated systematic review. Am J Prev Med. (2020) 59:123–36. 10.1016/j.amepre.2020.01.021 32418800 PMC7453837

[106] Samet JM Yoon S-Y Initiative WHOTF. Women and the tobacco epidemic: challenges for the 21st century. In:Jonathan M, editor. Geneva: World Health Organization. (2001).

[107] Ding D Gebel K Oldenburg BF Wan X Zhong X Novotny TE. An early-stage epidemic: a systematic review of correlates of smoking among Chinese women. Int J Behav Med. (2014) 21:653–61. 10.1007/s12529-013-9367-1 24222041 PMC4605608

[108] Okamoto J Sakuma KL Yan H Qiu P Palmer PH Johnson CA. qualitative exploration of youth in the “new” China: perspectives on tobacco use from adolescents in southwest China. Asia Pac J Public Health. (2012) 24:296–307. 10.1177/1010539510380735 20829274 PMC11353500

[109] Dawkins JC Hasking PA Boyes ME. Thoughts and beliefs about nonsuicidal self-injury: an application of social cognitive theory. J Am College Health. (2021) 69:428–34. 10.1080/07448481.2019.1679817 31689159

[110] Brandon TH Baker TB. The smoking consequences questionnaire: the subjective expected utility of smoking in college students. Psychol Assess J Consult Clin Psychol. (1991) 3:484–91. 10.1037/1040-3590.3.3.48418550294

[111] Lam CY Businelle MS Cofta-Woerpel L McClure JB Cinciripini PM Wetter DW. Positive smoking outcome expectancies mediate the relation between alcohol consumption and smoking urge among women during a quit attempt. Psychol Addict Behav. (2014) 28:163–72. 10.1037/a0034816 24731113 PMC5034867

[112] Chung T White HR Hipwell AE Stepp SD Loeber R. A parallel process model of the development of positive smoking expectancies and smoking behavior during early adolescence in Caucasian and African American girls. Addict Behav. (2010) 35:647–50. 10.1016/j.addbeh.2010.02.005 20188483 PMC2839038

[113] Khowaja LA Khuwaja AK Nayani P Jessani S Khowaja MP Khowaja S. Quit smoking for life—social marketing strategy for youth: a case for Pakistan. J Cancer Educ. (2010) 25:637–42. 10.1007/s13187-010-0088-8 20238199

[114] Feighery E Borzekowski DL Schooler C Flora J. Seeing, wanting, owning: the relationship between receptivity to tobacco marketing and smoking susceptibility in young people. Tob Control. (1998) 7:123–8. 10.1136/tc.7.2.123 9789929 PMC1759689

[115] Nurmansyah MI Umniyatun Y Jannah M Syiroj AT Hidayat DN. Knowledge, attitude and practice of cigarette smoking among senior secondary school students in Depok, Indonesia. Int J Adolescent Med Health. (2019) 33. 10.1515/ijamh-2018-0124 30913035

[116] Blecher E. The impact of tobacco advertising bans on consumption in developing countries. J Health Econ. (2008) 27:930–42. 10.1016/j.jhealeco.2008.02.010 18440661

[117] Fasbender U. Outcome expectancies. In:Zeigler-Hill V Shackelford TK, editors. Encyclopedia of Personality and Individual Differences. Cham: Springer International Publishing (2020). p. 3377–9.

[118] Baillie L Lovato CY Johnson JL Kalaw C. Smoking decisions from a teen perspective: a narrative study. Am J Health Behav. (2005) 29:99–106. 10.5993/AJHB.29.2.1 15698977

[119] Bidstrup PE Frederiksen K Siersma V Mortensen EL Ross L Vinther-Larsen M. Social-cognitive and school factors in lifetime smoking among adolescents. Canc Epidemiol Biomark Preven. (2008) 17:1862–71. 10.1158/1055-9965.EPI-07-2773 18708373

[120] ChoeE-Y 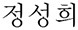 . The effect of self-efficacy promotion smoking cessation program on the amount of smoking, CO, urine cotinine level and self-efficacy for adolescent smokers. J Korean Biol Nurs Sci. (2012) 14:103–11. 10.7586/jkbns.2012.14.2.103

[121] Xiaoyi F Danhua L Chao F. Effects of perceived and actual partner smoking behavior on adolescent smoking behavior. Psychol Develop Educ. (2001) 2:2–30+5. Available online at: http://www.devpsy.com.cn/CN/Y2001/V17/I2/26

[122] Kishun J Kumar A Singh U. Correlates of cigarette smoking among adolescents in India. Indian J Community Med. (2021) 46:389–95. 10.4103/ijcm.IJCM_168_20 34759473 PMC8575211

[123] Tezera N Endalamaw A. Current cigarette smoking and its predictors among school-going adolescents in east africa: a systematic review and meta-analysis. Int J Pediatr. (2019) 2019:4769820. 10.1155/2019/4769820 31205474 PMC6530160

[124] Hawkins SS Bach N Baum CF. Impact of tobacco control policies on adolescent smoking. J Adolescent Health. (2016) 58:679–85. 10.1016/j.jadohealth.2016.02.014 27151762 PMC4877221

[125] China Moeotpsro,. Opinions on Further Strengthening the Work of Tobacco Control in Schools. (2010). Available online at: http://www.moe.gov.cn/srcsite/A17/moe_943/moe_946/201007/t20100713_92850.html (Accessed October 18, 2022).

[126] Ministry Ministry of Planning D Information Technology. Notice on Further Strengthening Tobacco Control Among Young People. (2019). Available online at: http://www.nhc.gov.cn/guihuaxxs/s7788/201911/53373070e15e45a5a30f3b7a037e05a4.shtml (accessed October 18, 2022).

[127] Notice on Further Strengthening the Building of Smoke-Free Schools. (2020). Available online at: http://www.nhc.gov.cn/guihuaxxs/s7788/202012/848defbba24c4fe98a02345a4dedde00.shtml (accessed October 18, 2022).

[128] Chinese Center for Disease Control Prevention. Results of Tobacco Prevalence Surveillance Among Middle School and College Students in China in 2021 were released. (2022). Available online at: https://www.chinacdc.cn/jkzt/sthd_3844/slhd_12885/202205/t20220529_259439.html (accessed October 18, 2022).

[129] Levy DT Chaloupka F Gitchell J. The effects of tobacco control policies on smoking rates: a tobacco control scorecard. J Public Health Manag Pract. (2004) 10:338–53. 10.1097/00124784-200407000-00011 15235381

[130] Niederdeppe J Farrelly MC Wenter D. Media advocacy, tobacco control policy change and teen smoking in Florida. Tob Control. (2007) 16:47–52. 10.1136/tc.2005.015289 17297073 PMC2598450

[131] Schmidt AM Kowitt SD Myers AE Goldstein AO. Attitudes towards potential new tobacco control regulations among US adults. Int J Environ Res Public Health. (2018) 15:72. 10.3390/ijerph15010072 29303963 PMC5800171

[132] Flor LS Reitsma MB Gupta V Ng M Gakidou E. The effects of tobacco control policies on global smoking prevalence. Nat Med. (2021) 27:239–43. 10.1038/s41591-020-01210-8 33479500 PMC7884287

[133] Méndez D Alshanqeety O Warner KE. The potential impact of smoking control policies on future global smoking trends. Tob Control. (2013) 22:46–51. 10.1136/tobaccocontrol-2011-050147 22535364

[134] Jayawardhana J Bolton HE Gaughan M. The association between school tobacco control policies and youth smoking behavior. Int J Behav Med. (2019) 26:658–64. 10.1007/s12529-019-09825-z 31741294

[135] Mannocci A Backhaus I D'Egidio V Federici A Villari P La Torre G. What public health strategies work to reduce the tobacco demand among young people? An umbrella review of systematic reviews and meta-analyses. Health Policy. (2019) 123:480–91. 10.1016/j.healthpol.2019.02.009 30922630

[136] Levy DT Tam J Kuo C Fong GT Chaloupka F. The impact of implementing tobacco control policies: the 2017 tobacco control policy scorecard. J Public Health Manag Pract. (2018) 24: 448–57. 10.1097/PHH.0000000000000780 29346189 PMC6050159

[137] Scalici F Schulz PJ. Parents' and peers' normative influence on adolescents' smoking: results from a Swiss-Italian sample of middle schools students. Sub Abuse Treat Preven Policy. (2017) 12:5. 10.1186/s13011-017-0089-2 28109189 PMC5251233

[138] Kunz JH Greenley RN Mussatto KA Roth-Wojcicki B Miller T Freeman ME. Personal attitudes, perceived social norms, and health-risk behavior among female adolescents with chronic medical conditions. J Health Psychol. (2014) 19:877–86. 10.1177/1359105313481077 23524992 PMC4540353

[139] Wong SL Shields M Leatherdale S Malaison E Hammond D. Assessment of validity of self-reported smoking status. Health Reports. (2012) 23:47–53. 22590805

