# Supplementary material for: Factors influencing adolescent experimental and current smoking behaviors based on social cognitive theory: A cross-sectional study in Xiamen
Source: Front Public Health. 2023 Mar 22;11:1093264. doi: 10.3389/fpubh.2023.1093264 (PMC10073720; doi:10.3389/fpubh.2023.1093264)
Supplement: Supplementary file 1 [file Table_1.DOCX]

**Supplement file**

**Statistical assumptions for multivariate logistic analysis and the checkout of this study.**

| **Statistical assumptions** | **Checkout** |
| --- | --- |
| 1. The dependent variable must be categorical variable with at least 1 independent variable, which can be either continuous or categorical. | The dependent variable (smoking behavior**)** was categorical variable (no-smoking, experimental smoking, and current smoking). There were more than 1 independent variables. |
| 2. Each observation is independent of each other. The classification of categorical variables (both dependent and independent variables) must be comprehensive and mutually exclusive between each classification. | Yes. |
| 3. The minimum sample size is required to be 15 times of the number of independent variables. | The number of independent variables was 26, and the minimum sample size is 390. There were 1937 participants (1937 > 390) in this study. |
| 4. There is a linear relationship between logit transformed values of continuous independent variables and the dependent variable. | Box-Tidwell method tests whether there is a linear relationship between the continuous independent variables and the logit transformed value of the dependent variable. The interaction items between the continuous independent variables and its natural logarithmic values are included in the regression equation. The interaction was not statistically significant. Therefore, there is a linear relationship between all the continuous independent variables and the logit transformed value of the dependent variable. |
| 5. There is no multicollinearity among the independent variables. | The correlation coefficient of any two independent variables is less than 0.7, in addition, and the variance inflation factor (VIF) is less than 10, indicating that there is no multicollinearity. |
| 6. There are no obvious outliers, leverage points and strong influence points. | Casewise Diagnostics tests with 2 standard deviations (SD) of standardized residual were used to indicate that there were no outliers. All the observed standardized residual were less than 2 SD in this study. SPSS did not output the Casewise List table. |
